# Failure to decrease HbA1c levels following TB treatment is associated with elevated Th1/Th17 CD4+ responses

**DOI:** 10.3389/fimmu.2023.1151528

**Published:** 2023-05-29

**Authors:** Robert Krause, Christian M. Warren, Joshua D. Simmons, Peter F. Rebeiro, Fernanda Maruri, Farina Karim, Timothy R. Sterling, John R. Koethe, Al Leslie, Yuri F. van der Heijden

**Affiliations:** ^1^ Africa Health Research Institute (AHRI), Durban, South Africa; ^2^ College of Health Sciences, School of Laboratory Medicine & Medical Sciences, University of KwaZulu Natal, Durban, South Africa; ^3^ Division of Infectious Diseases, Department of Medicine, Vanderbilt University School of Medicine, Nashville, TN, United States; ^4^ Department of Biostatistics, Vanderbilt University School of Medicine, Nashville, TN, United States; ^5^ Vanderbilt Tuberculosis Center, Vanderbilt University School of Medicine, Nashville, TN, United States; ^6^ Division of Infection and Immunity, University College London, London, United Kingdom; ^7^ The Aurum Institute, Johannesburg, South Africa

**Keywords:** hyperglycemia, tuberculosis, diabetes, HIV, HbA1c, IL-17, TNF-α, CX3CR1

## Abstract

**Introduction:**

The rising global burden of metabolic disease impacts the control of endemic tuberculosis (TB) in many regions, as persons with diabetes mellitus (DM) are up to three times more likely to develop active TB than those without DM. Active TB can also promote glucose intolerance during both acute infection and over a longer term, potentially driven by aspects of the immune response. Identifying patients likely to have persistent hyperglycemia following TB treatment would enable closer monitoring and care, and an improved understanding of underlying immunometabolic dysregulation.

**Methods:**

We measured the relationship of plasma cytokine levels, T cell phenotypes and functional responses with the change in hemoglobin A1c (HbA1c) before and after treatment of pulmonary TB in a prospective observational cohort in Durban, South Africa. Participants were stratified based on stable/increased HbA1c (n = 16) versus decreased HbA1c (n = 46) levels from treatment initiation to 12 month follow-up.

**Results:**

CD62 P-selectin was up- (1.5-fold) and IL-10 downregulated (0.85-fold) in plasma among individuals whose HbA1c remained stable/increased during TB treatment. This was accompanied by increased pro-inflammatory TB-specific IL-17 production (Th17). In addition, Th1 responses were upregulated in this group, including TNF-α production and CX3CR1 expression, with decreased IL-4 and IL-13 production. Finally, the TNF-α+ IFNγ+ CD8+ T cells were associated with stable/increased HbA1c. These changes were all significantly different in the stable/increased HbA1c relative to the decreased HbA1c group.

**Discussion:**

Overall, these data suggest that patients with stable/increased HbA1c had an increased pro-inflammatory state. Persistent inflammation and elevated T cell activity in individuals with unresolved dysglycemia following TB treatment may indicate failure to fully resolve infection or may promote persistent dysglycemia in these individuals, and further studies are needed to explore potential mechanisms.

## Introduction

The convergence of tuberculosis (TB) and type 2 diabetes mellitus (DM) threatens the life and livelihood of individuals in many resource-constrained settings. Concerns about the impact of DM on TB are particularly relevant in sub-Saharan Africa, which already bears over 80% of the world’s HIV-associated TB burden. The prevalence of DM is projected to rise steeply in the coming decades (1–3). An estimated 12.6% of the adult (age 20-79 years) sub-Sahara-African population have impaired glucose tolerance, 5.2% have type 2 diabetes, and over 50% of diabetes cases remain undiagnosed (4).

The interaction of TB and metabolic dysregulation is complex; while impaired glucose tolerance, including overt DM as well as prediabetes, increases the risk of active TB disease, the immune response to TB may also drive adverse changes in metabolic health (5–7). The increased risk of active TB in patients with DM has been attributed to multiple immunologic alterations, including monocyte and macrophage defects that contribute to delayed adaptive immune responses and altered *M. tuberculosis* (*Mtb)* specific pro- and anti-inflammatory T-helper type 1 (Th1) and Th17 responses (8, 9). Previous studies have also shown that patients with prediabetes and active TB have increased circulating levels of Th1, Th2, Th17, and regulatory cytokines (10, 11).

Although not all individuals with prediabetes go on to develop overt diabetes (12–15), the alterations in immune function in persons with prediabetes and DM that contribute to active TB disease overlap, in part, with studies linking immunologic biomarkers to the development of metabolic disease in the absence of infectious conditions. In the general population, a reduced proportion of naïve and regulatory (T_reg_) CD4+ T cells (16–18), higher circulating memory CD4+ T cells (16, 19), and a shift towards pro-inflammatory Th1 and Th17 helper cells (18) was associated with prevalent diabetes. Taken together, these findings suggest a potential bidirectional relationship in which altered immunity in glucose intolerance predisposes to active TB disease, which subsequently may worsen metabolic function.

The potential for an immune response against infectious conditions to promote metabolic dysregulation has been investigated in persons with HIV (PWH) (20, 21), but there are fewer data among individuals with TB. We and others, have previously demonstrated that elevation of soluble receptor of tumor necrosis factor (TNF)-α-1 (sTNFR1) and interleukin-6 (IL-6), and increased expression of CD45RO and CD57 on CD4+ T cells, are associated with increased incidence of DM among PWH (16, 22, 23). Additionally, higher baseline frequencies of CD4+ T effector memory RA+ (T_EMRA_) cells (CD45RA+ CD27-) and senescent T cells (CD4+ CD28-) have been associated with incident diabetes in PWH (21). It is unclear, however, if these factors are also associated with DM in patients with TB disease.

In this study we sought to build upon the immunometabolism model linking alterations in adaptive immunity to the development of DM to explore impaired glucose tolerance (hemoglobin A1c [HbA1c] ≥5.7%) in TB disease using longitudinal data from drug-susceptible TB patients in South Africa. We assessed a broad range of innate and adaptive immune markers among individuals undergoing treatment for active TB to identify factors associated with the persistence or worsening of glucose intolerance.

## Methods

### Study population

Participants enrolled in the Africa Health Research Institute (AHRI) site in Durban, South Africa of the Regional Prospective Observational Research in Tuberculosis consortium of South Africa (RePORT SA) were eligible for inclusion. All participants included in the study were individuals with pulmonary, drug-susceptible, culture-confirmed *Mycobacterium tuberculosis* (*Mtb*). Study enrollment opened in December 2017 and closed in November 2019. Individual baseline measures of weight (kg), height (m), body mass index (BMI), HbA1c (%), age (years), sex (self-reported, male or female), HIV status (by Western Blot and PCR, positive or negative) as well as antiretroviral therapy (ART) use, and blood samples (CD4+ T-lymphocyte counts) were collected at the time of treatment initiation (baseline); HbA1c and CD4+ counts were measured again at 12-15 months (follow-up). HbA1c levels were defined as normal (<5.7%), prediabetes (5.7-6.4%), and diabetes (≥6.5%), with the HbA1c characteristics of the study population at baseline and follow up summarized in **Supplementary Table 1**. These HbA1c based definitions are based on the American Diabetes Association guidelines (24).

This study was approved by the Vanderbilt Institutional Review Board (IRB#191176). Informed consent was obtained under the Biomedical Research Ethics Committee (BREC) at the University of Kwazulu-Natal (UKZN) Ref No: BE423/19, and the RePORT South Africa study (IRB#160918) at Vanderbilt.

### T-lymphocyte stimulation conditions and flow cytometry

Peripheral blood mononuclear cells (PBMC) were isolated by density gradient centrifugation through Histopaque 1077 (SIGMA) according to standard procedure. To induce cytokine production, 10^6^ PBMC each were stimulated for 6 hours with the mitogens PMA and ionomycin or *Mtb* peptides (MTB300) or left unstimulated. Cells were then washed in PBS and surface stained in a 25 µl antibody mix containing a Live/Dead™ fixable Aqua-dead cell staining reagent (1:200 dilution, Invitrogen, Carlsbad, CA, USA). Combinations of the surface antibodies as listed in **Supplementary Table 2** or **3** from BD Biosciences (Franklin Lakes, NJ, USA) or from BioLegend (San Diego, CA, USA). *Ex vivo* surface marker staining was completed for 20 min in the dark at room temperature (RT), followed by two washes with PBS. Cells were then fixed in 2% PFA ready for acquisition. Alternatively for intracellular cytokine staining (ICS) the cells were permeabilized, following surface marker staining, with 100 µl BD FixPerm at 4°C for 20 min, washed twice with BD PermWash buffer and blocked with 100 µl of a 20% goat serum PBS solution. Finally, cells were stained with a 25 µl cytokine antibody mix for 20 min at RT, washed with PermWash buffer and suspended in 2% PFA. Cells were acquired on a FACSAria Fusion III flow cytometer (BD), and data analyzed with FlowJo version 9.9.6 software (Tree Star).

### T-lymphocyte cluster determinations

An unsupervised analysis workflow that includes dimension reduction and clustering was applied to each identified cell type (CD4+ and CD8+) from each cytometric experiment (ICS and *ex vivo*) independently. The workflow was executed using the R programming language version 4.1. To start, FCS files were down sampled to 5000 events with seed set to 123 for reproducibility. The down sampled data was then concatenated and transformed (25). The flowjo_biexp function from the flowWorkspace (26) package was used to transform the data. Transformation parameters, such as maxValue, pos, neg, and widthBasis were parsed from a FlowJo workspace XML file. The XML file was exported from the same workspace where the FCS files were previously gated for CD4+ T and CD8+ T cells and other known populations. The HarmonyMatrix function from the harmony package (27) was then used to help mitigate any batch effects. The do_pca argument was set to FALSE and the biexponential-transformed data was used as the input embedding on which the harmony algorithm would act directly. Uniform Manifold Approximation and Projection (UMAP) was used to facilitate visualization of the corrected, multidimensional space. This was done with the umap function from the uwot package (28) with nearest neighbors (n_neighbors) set to 30. Data points were initialized (init) using the “spca” setting and effective minimum distance between embedded points (min_dist) was set to 0.1. The FlowSOM function from the FlowSOM package (29) was used to iteratively cluster the multidimensional, batch-corrected data. A range of 3 to 25 clusters was tested with seed set to 123. Heatmaps representative of each cluster’s phenotype were produced per iteration (30). These heatmaps were used to guide the selection of an appropriate number of meta-clusters (nClus). For the CD4+ T cell ICS experiment analysis, a cluster number of 10 was chosen. A cluster number of 9 was chosen for each of the other three data sets analyzed (CD8+ T cell ICS, CD4+ T cell ex vivo, and CD8+ T cell ex vivo analyses). Cluster frequencies were calculated and used for downstream statistical testing.

### Plasma cytokine and CMV serostatus assays

Patient plasma cytokine levels were measured using the Thermo Fisher human inflammation panel 20 Plex kit (cat. no. EPX200-12185-901) and the Luminex Bio-Rad Bio-Plex™ 200 system. To control for the effect of CMV infection, which often elicits Th1 biased T cell responses (20, 31), the patient CMV serostatus was assessed by the GenWay CMV IgG ELISA test kit (cat. no. GWB-892399).

### Outcome

The primary outcome in these analyses was individual follow-up HbA1c measure in relation to baseline HbA1c; the HbA1c outcome was dichotomized as decreased (follow-up measure < baseline measure) or stable/increased (follow-up measure ≥ baseline measure).

### Statistical analysis

Participant demographic and clinical characteristics were compared by HbA1c change over time (decreased versus stable/increased) using Chi-square tests for categorical variables and Kruskall-Wallis tests for continuous variables. Differences in the percentages of CD4+ cells of particular phenotypes (percent of individuals’ total CD4+ population, defined by surface receptor and cytokine characteristics) were also examined across outcome categories, comparing follow-up to baseline measures, under multiple stimulation conditions. These differences were assessed for each phenotype under each stimulation condition both by Kruskal-Wallis test, and if considered of potential interest for hypothesis generation (p<0.10), by linear regression, adjusting for baseline HbA1c measure. All analyses were conducted in Stata 15.1 (StataCorp, College Station, TX). Family-wise error rate corrections were not applied for multiple testing, as hypothesis tests for specific markers were determined *a priori* as per Kumar et al. (11), but Bonferroni-corrected p-values are provided in the footnotes of each table for the reader’s consideration.

## Results

Of 62 total participants, approximately 1/3 (n = 23) had normal HbA1c levels at baseline, half (n = 33) had prediabetes, and the remainder (n = 6) were categorized as having diabetes (24). Three of the six patients with HbA1c ≥6.5% at baseline self-reported DM. The HbA1c levels of the study population are summarized in **Supplementary Table 1**. The HbA1c levels for the prediabetes and diabetes groups tended to decrease following treatment, with the prediabetes group showing a significant decrease (p < 0.0001) in baseline to follow-up HbA1c values. Since the HbA1c levels varied considerably at baseline versus follow-up, we separated participants into those with any increased (n = 10) or decreased (n = 46) follow-up HbA1c relative to baseline. We did not implement a threshold to qualify an alteration as increased or decreased, rather any change was considered. Participants without change in HbA1c (n = 6) were included in the increased HbA1c group; four of the six had normal HbA1c.

**Table 1** compares the population demographics/characteristics of the two groups. Neither group differed significantly by age, sex, or HIV status. All 62 participants were CMV IgG positive. Most patients (n = 51 [82%]) were cured or had treatment completion; seven had treatment failure, three did not complete treatment, and one had relapse after cure. Due to our small study sample size and since several studies have investigated the associated factors for progression from prediabetes to diabetes or reversion to normoglycemia (12–15), we focused on measuring immune-associated changes relating to HbA1c fluctuations following TB treatment and did not consider participants with overt diabetes as a separate group.

**Table 1:** Study population characteristics (N=62).

	Decreased-HbA1c (n=46)		Stable/increased-HbA1c (n=16)		
Characteristic	N	%	N	%	p-value*
Age, years**	33.5	(27, 43)	37	(31.5, 41.5)	0.53
Sex					0.31
Male	28	60.9	12	75	
Female	18	39.1	4	25	
HIV-Status					0.57
Negative	18	39.1	5	31.2	
Positive	28	60.9	11	68.8	
HIV-Positive, ART-Receipt					0.51
No	16/28	57.1	5/11	45.5	
Yes	12/28	42.9	6/11	54.5	
HbA1c at Baseline**	6	(5.7, 6.2)	5.3	(5.1, 5.6)	
HbA1c at Follow-up**	5.5	(5.3, 5.8)	5.5	(5.2, 5.6)	

*X^2^ test for categorical variables; Kruskall-Wallis test for continuous variables **Continuous variables presented as “median (interquartile range)”

First, we assessed plasma cytokine levels of all participants to determine if changes in HbA1c following TB treatment were associated with changes in systemic markers of inflammation (**Table 2**). Only two markers showed significant changes between study groups. CD62 P-selectin, a marker of chronic inflammation (32), was significantly higher in the patients with stable/increased HbA1c following treatment relative to those in whom HbA1c was reduced. Conversely, the anti-inflammatory cytokine IL-10 was significantly reduced in participants with stable/increased HbA1c but remained unchanged in patients with decreased HbA1c. Although no significant differences were observed for the other soluble factors measured, including proinflammatory markers such as IL-6 and IL-1b, these data suggested an association between increasing HbA1c and a more proinflammatory state following TB treatment.

**Table 2:** Comparison of Fold-change (follow-up vs Baseline) in plasma cytokine levels relative to HbA1c change during follow-up (increase vs. decrease), (N=62).

	Decreased HbA1c		Stable/increased HbA1c		
Marker	Median fold change	IQR	Median fold change	IQR	p-value*
IFN alpha	0.92	(0.76, 1.1)	0.97	(0.81, 1.1)	0.47
**IL-10**	**1**	**(0.89, 1.3)**	**0.85**	**(0.76, 1)**	**0.04**
IL-13	0.99	(0.77, 1.2)	0.88	(0.83, 1.1)	0.35
IL-4	0.96	(0.81, 1.2)	0.92	(0.81, 1.2)	0.94
CD62E (E-selectin)	0.51	(0.32, 0.94)	0.63	(0.34, 0.98)	0.68
**CD62P (P-selectin)**	**0.67**	**(0.45, 1.2)**	**1.5**	**(0.79, 2.2)**	**0.01**
GM-CSF	1	(0.89, 1.2)	0.88	(0.77, 0.98)	0.06
IFN gamma	0.93	(0.71, 1.1)	0.84	(0.7, 0.96)	0.3
IL1 alpha	0.89	(0.78, 1)	0.77	(0.67, 1.1)	0.3
IL1 beta	0.98	(0.77, 1.2)	0.95	(0.79, 1.2)	0.75
IL-12p70	0.88	(0.73, 1.1)	0.85	(0.7, 1)	0.88
IL-17A (CTLA-8)	0.98	(0.75, 1.2)	0.86	(0.72, 0.97)	0.14
IL-6	0.93	(0.79, 1)	0.83	(0.72, 0.99)	0.52
IL-8 (CXCL8)	0.74	(0.47, 0.88)	0.77	(0.54, 0.97)	0.54
IP-10 (CXCL10)	0.27	(0.07, 0.64)	0.4	(0.18, 0.71)	0.52
MCP1 (CCL2)	1.1	(0.83, 1.3)	1.3	(0.79, 1.7)	0.44
MIP1 alpha (CCL3)	0.79	(0.67, 1)	0.85	(0.76, 1.1)	0.33
MIP1 beta (CCL4)	0.93	(0.69, 1.1)	0.96	(0.78, 1.1)	0.72
ICAM-1	0.82	(0.62, 0.99)	0.89	(0.61, 1.1)	0.37
TNF alpha	1	(0.78, 1.1)	0.95	(0.89, 0.61)	0.61

*X^2^ test for categorical variables; Kruskall-Wallis test for continuous variables. Continuous variables presented as “median fold change (interquartile range)” percentages. Bold proportions/distributions are significantly different (p<0.05). Bonferroni-corrected threshold for multiple testing is p=0.0025.

**Figure 1:**
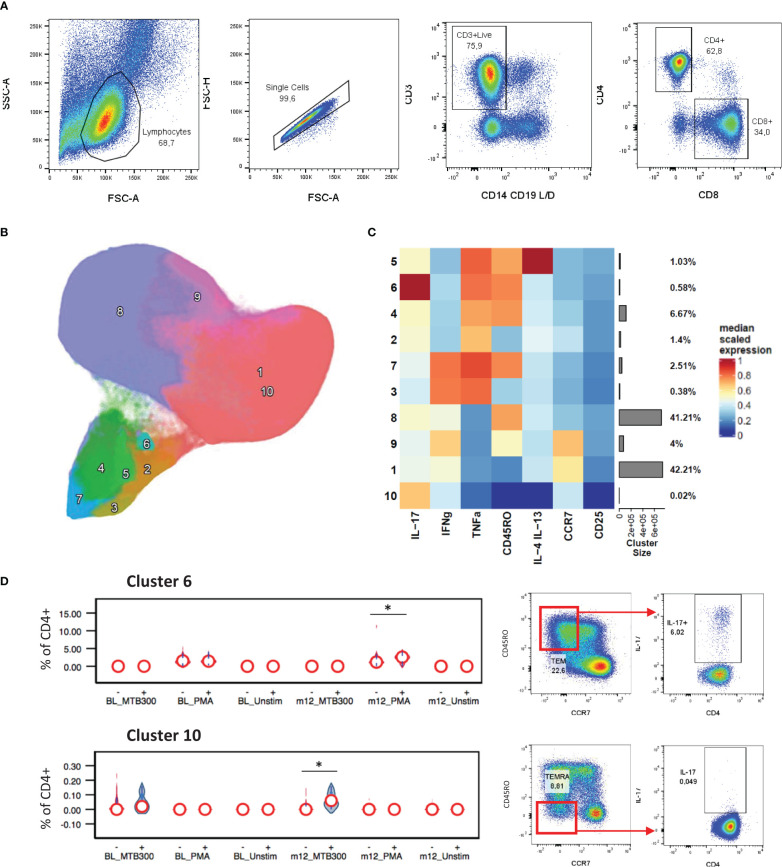
CD4+ cytokine profiles associated with HbA1c levels. General gating strategy to identify CD4+ and CD8+ T cells **(A)**. UMAP projection of the 10 FlowSOM generated CD4+ T cell clusters **(B)**. The resulting cluster frequency and median fluorescence intensity of detected markers expressed by each cluster were presented as a heatmap **(C)**. Clusters 6 and 10 significantly associated with stable/increased HbA1c category **(D)**. Participant groups with increased or decreased HbA1c levels were represented with “+” or “-” respectively. Respective flow cytometry dot plots were included alongside, displaying IL-17 production. Statistical analyses were done using Kruskal-Wallis test and P values are denoted as * < 0.05.

To determine if stable/increased HbA1c levels were associated with differences in T cell immunity, PBMC samples were stimulated as described and an unbiased clustering approach used to identify CD4+ or CD8+ T cell phenotypes of interest (**Figure 1A**). The main cytokine producing CD4+ populations, clusters 2-7 (**Figure 1B**), all grouped together and displayed either an effector memory (CD45RO+ CCR7-) or a TEMRA (CD45RO- CCR7-) phenotype. This approach identified a PMA-stimulated IL-17+ TNF-α+ CD4+ cluster (cluster 6) which was positively associated with HbA1c levels (**Figures 1C, D**). At follow-up after TB treatment, the capacity of CD4+ T cells to produce IL-17 was increased in patients with stable/increased HbA1c. This population has an effector memory phenotype (CD45RO+ CCR7-) and comprises about 0.6% of total CD4+ T cells. In addition, *Mtb*-specific T cells, which produced IL-17 but not TNF-α+ following stimulation with MTB300, were also significantly elevated in participants who had stable/increased HbA1c. These cells had a TEMRA (CD45RO- CCR7-) phenotype. To confirm these differences identified using an unbiased approach, we gated on total cytokine production for CD4+ T cells using conventional Boolean gating (**Table 3**; **Supplementary Figure 1**). Despite seeing no difference in plasma TNF-α, this revealed a significant increase in TNF-α production from CD4+ T cells stimulated with MTB300 antigen in patients with stable/increased HbA1c. Together these data suggest that stable/increased HbA1c following TB treatment was associated with elevated Th1 and Th17 CD4+ responses. In contrast, the capacity for Th2 responses (based on the production of IL-4/-13) was reduced in participants with stable/increased HbA1c, reaching significance at follow-up with PMA stimulation (**Supplementary Table 4**).

**Table 3:** Manual gating of total cytokine production by CD4+ T cells.

	Decreased-HbA1c		Stable/increased-HbA1c		
Characteristic	Median %	IQR	Median %	IQR	p-value*
TNF-α+ Only, BL+ PMA	20	(12, 27)	19	(11, 28)	0.93
TNF-α+ Only, BL+ TB300	**0.011**	**(0, 0.027)**	**0.05**	**(0.012, 0.073)**	**0.03**
TNF-α+ Only, BL+ Unstim	0	(0, 0.02)	0.005	(0, 0.031)	0.23
TNF-α+ Only, m12+ PMA	25	(18, 30)	23	(18, 32)	0.85
TNF-α+ Only, m12+ TB300	**0.008**	**(0, 0.015)**	**0.029**	**(0.005, 0.041)**	**0.01**
TNF-α+ Only, m12+ Unstim	0.001	(0, 0.013)	0.011	(0, 0.03)	0.12

*χ2 test for categorical variables; Kruskall-Wallis test for continuous variables. Continuous variables presented as “median (interquartile range)” percentages. Bold proportions/distributions are significantly different (p<0.05). Bonferroni-corrected threshold for multiple testing is p=0.008.

**Figure 2:**
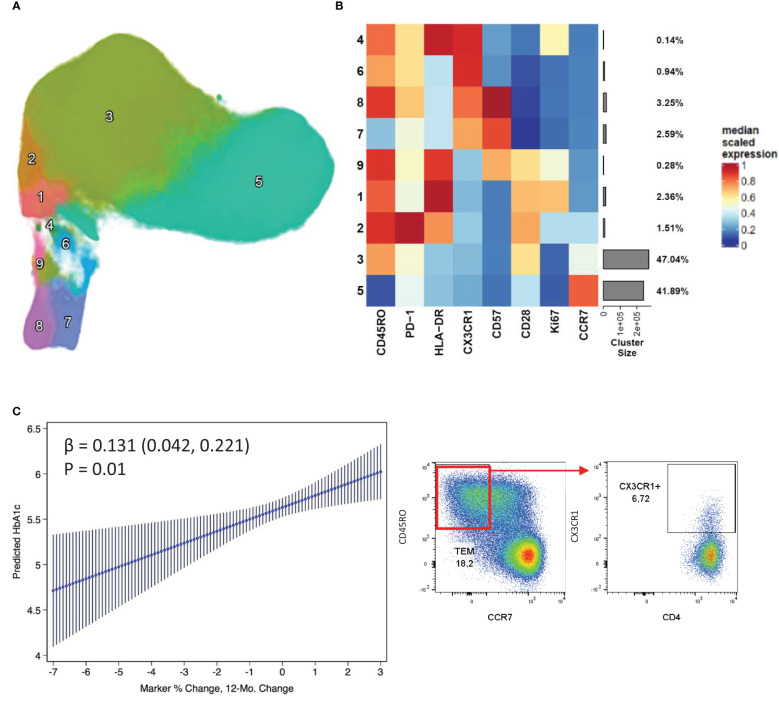
CD4+ CX3CR1 surface expression associated with increased HbA1c change. Nine distinct clusters were identified by FlowSOM and projected as a UMAP **(A)**. The cluster frequency and marker expression intensity were presented as a heatmap **(B)**. Linear regression of a CX3CR1 expressing cluster 6 which associated positively with stable/increased HbA1c **(C)**. Respective flow cytometry dot plots were included alongside, displaying CX3CR1 expression.

An additional phenotyping flow panel was used, and unbiased clustering revealed distinct clusters suggestive of activated and exhausted T cells (based on the expression of HLA-DR, PD-1, CD57 and CX3CR1) (**Figures 2A, B**). Of these, cluster 6, distinguished by expression of CX3CR1 in the absence of HLA-DR or CD57, showed a positive association with stable/increased HbA1c (**Figure 2C**). Furthermore, looking at total CX3CR1+ CD4+ T cells, their frequency was higher at baseline and follow-up in the patients with stable/increased HbA1c (~1.4 fold higher). In addition, cluster 8 (CD57+) almost doubled in frequency from baseline to follow-up for patients with stable/increased HbA1c, but this did not reach statistical significance.

**Figure 3:**
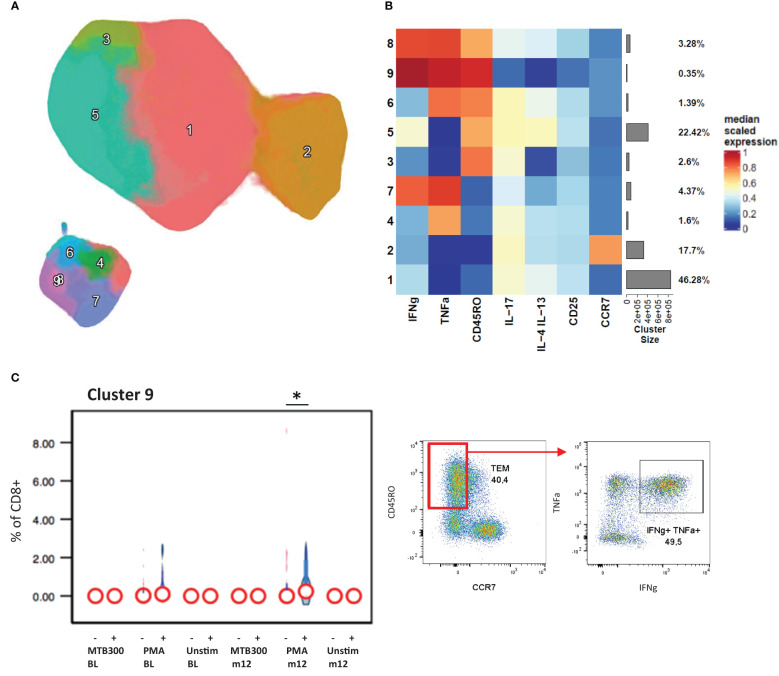
CD8+ cytokine profiles associated with HbA1c levels. The CD8+ T cells clustered into 9 distinct populations by FlowSOM analysis and displayed by UMAP projection **(A)**. The cluster frequency and marker expression intensity were compared in the resulting heatmap **(B)**. Cluster 9 associated significantly with stable/increased HbA1c category **(C)**. Participant groups with increased or decreased HbA1c levels were represented with “+” or “-” respectively. Respective flow cytometry dot plots were included alongside, displaying IFNg and TNFα co-expression. Statistical analyses were done using Kruskal-Wallis test and P values are denoted as * < 0.05.

**Figure 4:**
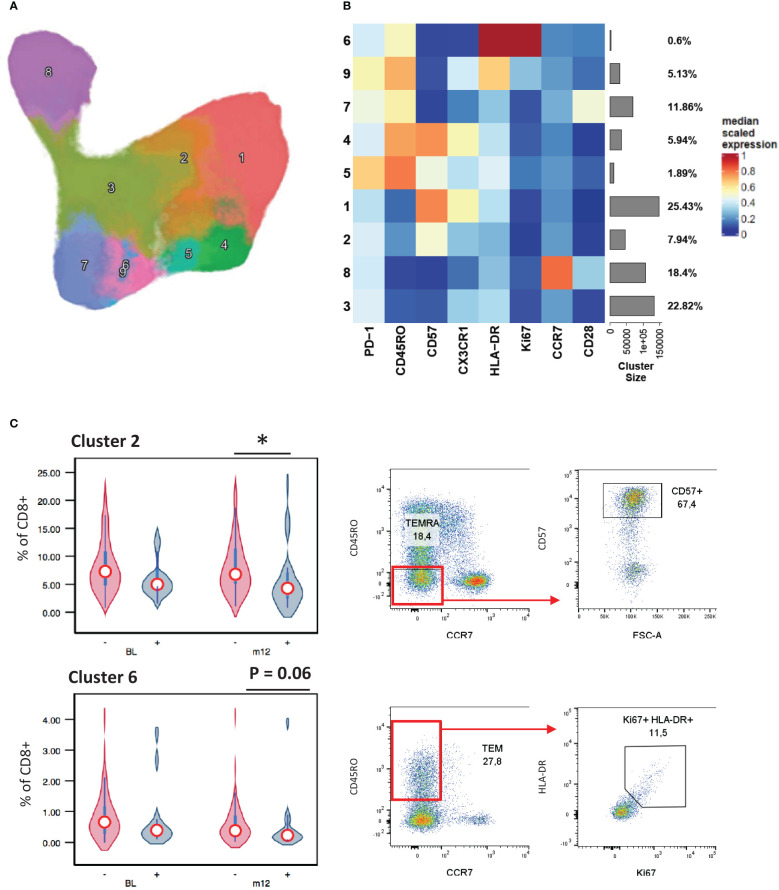
CD8+ surface marker expression associated with HbA1c levels. A UMAP projection of the 9 unique CD8+ T cell clusters identified by FlowSOM analysis **(A)**. The population frequencies and their relative marker expression levels were summarized in a heatmap **(B)**. Cluster 2 associated stable/increased with HbA1c category **(C)**. Participant groups with increased or decreased HbA1c levels were represented with “+” or “-” respectively. Respective flow cytometry dot plots were included alongside, displaying CD57+ TEMRA and Ki67 and HLA-DR co-expressing TEM. Statistical analyses were done using Kruskal-Wallis test and P values are denoted as * < 0.05.

Examining CD8+ T cell responses in the same way we found that a PMA stimulated IFNγ+ TNF-α+ effector memory CD8+ T cell population (cluster 9) was upregulated in patients with stable/increased HbA1c at follow-up (**Figure 3**). As expected, the IL-17, IL-4 and IL-13 expression were confined to CD4+ T cells, with little or no detection in CD8+ populations. From the phenotyping panel (**Figure 4**), cluster 2, consistent with CD8+ TEMRA CD57+ population, was lower in patients with stable/increased HbA1c at both baseline and follow-up but reached significance at follow-up (**Figure 4C**). A similar trend, although not significant, was observed for cluster 6, which expressed markers of activation and proliferation (HLA-DR+ Ki67+).

## Discussion

In our drug-susceptible TB study cohort with high proportion of PWH, we investigated the immune factors associated with persistent glucose intolerance following treatment of active disease. Longitudinal sampling allowed us to group patients based on stable/increased HbA1c or decreased HbA1c. Stable/increased HbA1c one year after TB treatment initiation was associated with a pro-inflammatory state reflected by elevated plasma cytokines, Th1 (CX3CR1+) and Th17 CD4+ T cell responses.

Six patients had an HbA1c ≥6.5% at baseline, three of whom received anti-diabetic medications, though they did not start these medications during follow up. Despite a relatively low proportion of patients with DM in our cohort, over half (56%) had HbA1c levels consistent with prediabetes at baseline. We found that most patients had resolution of hyperglycemia after TB treatment, consistent with well-documented transient hyperglycemia (33). It is unclear whether the hyperglycemia observed in our study and others increases long-term risks for development of DM (34).

Although most participants did not have baseline HbA1c levels indicative of overt DM, and most had resolution of hyperglycemia, those with increased HbA1c levels tended to display a more pro-inflammatory state characterized by elevated CD62 P-selectin and depressed IL-10 plasma levels. Although less pronounced, likely due to the small sample size, this is in keeping with similar trends observed by Randeria et al. who compared type 2 diabetics (HIV and TB negative) to healthy controls (35). HIV and TB co-infection as well as the effect of ART may have influenced our findings. Nonetheless we observed elevated soluble CD62 P-selectin, which serves as an indicator of vascular inflammation and is characteristic of type 2 diabetes (36–38). Several studies have described either an increased expression of IL-10 in plasma of persons with type 2 diabetes (35) or unchanged/reduced levels (39, 40) relative to healthy controls. Interestingly, glucose intolerance reduces IL-10 signaling efficiency, resulting in increased TNF-α production (39).

Although we did not observe a difference in TNF-α, we did detect reduced IL-10 plasma levels. Reduced IL-10 signaling has been associated with type 2 diabetes (40). Together the lower IL-10 levels and reduced signaling tie in well with a pro-inflammatory T cell response. In this regard, participants with HbA1c levels that increased or remained stable over time had an increased capacity of CD4+ T cells to produce IL-17 and TNFα (following PMA stimulation), and for *Mtb*-specific CD4+ T cells to produce IL-17 alone following stimulation with the TB-antigen pool MTB300 (41). Recent data from a human TST challenge model suggest that TB-specific Th17 T cell responses are associated with TB pathogenesis (42). Although most participants had successful treatment and were culture negative at the end of TB therapy, it is possible that incomplete Mtb killing may occur within the lungs of some TB patients, as demonstrated in human studies using direct sampling of the lung environment (43). If so, then residual infection might be expected to prevent the resolution of HbA1c and be associated with persistent inflammation and pathogenic IL-17 T cell responses. Regardless, dysglycemia is associated with decreased Th1 and Th17 responses to *Mtb* antigens in individuals with latent TB infection (44, 45), suggesting a different relationship following TB treatment. Interestingly, the depression of these responses was linked to elevated IL-10, which was significantly lower in individuals following treatment who had stable or increased HbA1c levels, supporting this hypothesis.

In addition, we observed an association between CD4+ T cell expression of CX3CR1, a marker associated with Th1 effector populations (46, 47), and stable/increased HbA1c levels. Lau et al. also found DM was associated with an increase in CX3CR1 expressing T cells and a reduced Th2 response (48). The CX3CR1-fractalkine axis mediates the migration of CX3CR1 expressing lymphocytes to inflamed tissue endothelium (46, 49). These T cells often have anti-viral and cytotoxic properties and are associated with CMV and HIV infection (31, 50). All participants in this study had CMV reactive plasma and 69% of participants in the stable/increased HbA1c group were HIV positive, therefore this increased CX3CR1+ CD4+ T cell frequency is also consistent with their anti-viral properties. Nonetheless, CX3CR1 expression on CD4+ T cells associated positively with stable/increased HbA1c. This, in turn, is consistent with this phenotype’s association with the progression of metabolic disease (20).

Our study had limitations. First, our sample size was small. Despite the small number of patients with DM at baseline, we were able to demonstrate the association of stable/increased HbA1c with elevated Th1 and Th17 CD4+ responses, providing direction for further study. Second, we had a single follow-up measurement of HbA1c and CD4+ cells 12-15 months after TB treatment initiation. Although this timeframe allowed time for a complete course of treatment, it remains unclear what risk, if any, is conferred by the dysregulation we observed in those whose HbA1c remained stable or increased. Finally, our investigations were exploratory and need to be confirmed in additional contexts and populations.

## Conclusion

Identifying markers that associate with glucose intolerance could help identify metabolically fragile individuals who need closer monitoring/treatment. Monitoring the pro-inflammatory T cell response in hyperglycemic patients after TB treatment could prove useful in this regard. Further work investigating the mechanisms linking these immune pathways to metabolic dysregulation and poor clinical outcomes are needed.

## Data availability statement

The original contributions presented in the study are included in the article/**Supplementary Material**. Further inquiries can be directed to the corresponding author.

## Ethics statement

This study was approved by the Vanderbilt Institutional Review Board (IRB#191176). Informed consent was obtained under the Biomedical Research Ethics Committee (BREC) at the University of Kwazulu-Natal (UKZN) Ref No: BE423/19, and the RePORT South Africa study (IRB#160918) at Vanderbilt. The patients/participants provided their written informed consent to participate in this study.

## Author contributions

RK, CW and JS share co-first authorship. RK conducted experimental work, analysis and wrote the manuscript. CW contributed to analysis and writing. JS contributed to analysis and writing. PR contributed to statistical analysis and writing. FM, FK and TS contributed to study design, administration and writing. JK contributed to study design, analysis and writing. AL contributed to study design and writing. YV contributed to study design, funding, analysis and writing. All authors contributed to the article and approved the submitted version.
