# Supplementary material for: Failure to decrease HbA1c levels following TB treatment is associated with elevated Th1/Th17 CD4+ responses
Source: Front Immunol. 2023 May 29;14:1151528. doi: 10.3389/fimmu.2023.1151528 (PMC10258338; doi:10.3389/fimmu.2023.1151528)
Supplement: Supplementary file 1 [file DataSheet_1.pdf]

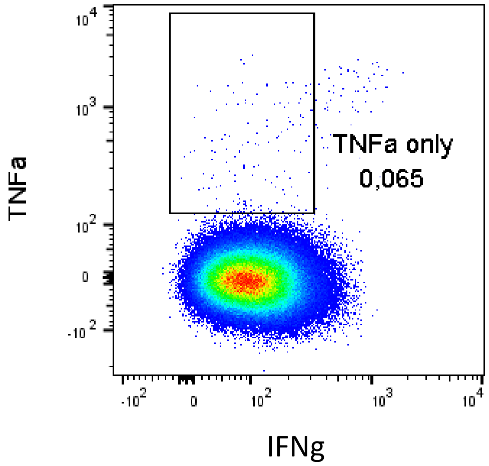

Supplementary Figure 1: Representative flow plot of CD4+ TNFa+ population presented in Table 3.

Supplementary Table 1. HbA1c characteristics of the study population (N=62)

	Normal (<5.7%)		Pre-diabetic (5.7-6.4%)		Diabetic (≥6.5%)	
	**BL**	**m12**	**BL**	**m12**	**BL**	**m12**
**Median**	5.3	5.3	6	5.5	8.0	6.9
**IQR**	(5.2, 5.5)	(5.1, 5.5)	(5.9, 6.2)	(5.3, 5.6)	(6.7, 9.5)	(5.8, 8.1)
**p-value***	0.73		<0.0001		0.09	
**N**	23		33		6	

*Wilcoxon matched pairs signed rank test.

Supplementary Table 2. Surface marker panel

Marker	Fluor	Clone	Cat. #	Manufacturer
CD14	Hv500	M5E2	561392	BD
CD19	Hv500	HIB19	561125	BD
Aqua Live/Dead	Hv500		L34957	Invitrogen
CD3	Bv786	SK7	563800	BD
CD4	PerCP-Cy5.5	RPA-T4	560650	BD
CD8	AF700	RPA-T8	557945	BD
CX3CR1	PE	2A9-1	565798	BD
PD-1	Bv421	EH12-2H7	562516	BD
CD57	FITC	NK-1	561906	BD
Ki67	PE-Cy7	Ki67	350526	BioLegend
HLA-DR	APC-Cy7	L243	307617	BioLegend
CCR7	APC	G043H7	353214	BioLegend
CD45RO	PECF594	UCHL1	562299	BD
CD28	Bv711	CD28.2	302948	BioLegend

Supplementary Table 3. ICS marker panel

Marker	Fluor	Clone	Cat. #	Manufacturer
IFNγ	PE-Cy7	4S.B3	502528	BioLegend
IL-4	Bv421	MP4-2502	500825	BioLegend
IL-13	Bv421	JES10-5A2	501916	BioLegend
IL-17A	PE	TC11-18H10.1	506938	BioLegend
TNF-α	AF700	MAb11	557996	BD
CD45RO	PECF594	UCHL1	562299	BD
CD3	APC-Cy7	SK7	344840	BioLegend
CD4	PerCPCy5.5	RPA-T4	560650	BD
CD8	DAPI	RPA-T8	563795	BD
CD25	BB515 (FITC)	M-A251	565096	BD
CCR7	APC	G043H7	353214	BioLegend
CD14	Hv500	M5E2	561392	BD
CD19	Hv500	HIB19	561125	BD
Aqua Live/Dead	Hv500		L34957	Invitrogen

Supplementary Table 4. Manual gating of total cytokine production by CD4+ T cells

	Decreased-HbA1c		Stable/increased-HbA1c		
**Characteristic**	**Median %**	**IQR**	**Median %**	**IQR**	**p-value***
IFNγ+ Only, BL+ PMA	0.062	(0.012, 0.26)	0.067	(0.013, 1.6)	0.61
IFNγ+ Only, BL+ TB300	0.014	(0, 0.19)	0.015	(0, 1.2)	0.99
IFNγ+ Only, BL+ Unstim	0.043	(0.003, 0.46)	0.001	(0, 5.3)	0.17
IFNγ+ Only, m12+ PMA	0.030	(0.005, 0.13)	0.040	(0.029, 0.19)	0.19
IFNγ+ Only, m12+ TB300	0.004	(0, 0.111)	0.008	(0.001, 0.048)	0.67
IFNγ+ Only, m12+ Unstim	0.023	(0, 0.12)	0.074	(0.003, 0.21)	0.27
IL4+/IL13+, BL+ PMA	2.65	(1.12, 4.17)	1.74	(0.9, 3.54)	0.39
IL4+/IL13+, BL+ TB300	0.07	(0.03, 0.19)	0.07	(0.03, 0.14)	0.49
IL4+/IL13+, BL+ Unstim	0.15	(0.05, 0.39)	0.06	(0.02, 0.19)	0.75
IL4+/IL13+, m12+ PMA	2.98	(1.09, 4.56)	2.24	(0.98, 4.14)	0.01
IL4+/IL13+, m12+ TB300	0.08	(0.01, 0.2)	0.07	(0.04, 0.11)	0.26
IL4+/IL13+, m12+ Unstim	0.11	(0.03, 0.36)	0.11	(0.07, 0.21)	0.47
IL17+, BL+ PMA	1.70	(0.97, 2.58)	1.61	(1.07, 2.68)	0.79
IL17+, BL+ TB300	0.03	(0.01, 0.08)	0.02	(0.01, 0.03)	0.66
IL17+, BL+ Unstim	0.02	(0, 0.13)	0.02	(0.01, 0.03)	0.36
IL17+, m12+ PMA	1.29	(0.86, 2.04)	2.48	(1.74, 3.59)	0.16
IL17+, m12+ TB300	0.01	(0, 0.04)	0.02	(0.01, 0.03)	0.30
IL17+, m12+ Unstim	0.02	(0.01, 0.08)	0.02	(0.02, 0.03)	0.09

*χ^2 test for categorical variables; Kruskall-Wallis test for continuous variables. Continuous variables presented as "median (interquartile range)" percentages. Bold proportions/distributions are significantly different ($p<0.05$).
